# Lipid Accumulation Product Index as a Marker of Metabolic Syndrome in Women With Polycystic Ovary Syndrome: A Systematic Review and Meta‐Analysis

**DOI:** 10.1002/edm2.70078

**Published:** 2025-09-04

**Authors:** Seyed Arsalan Seyedi, Seyed Ali Nabipoorashrafi, Samira Amin Afshari, Afsoun Mansouri, Dorsa Alizadegan, Sara Hobaby, Fatemeh Esmaeilpur Abianeh, Jeffrey I. Mechanick, Alireza Esteghamati

**Affiliations:** ^1^ Endocrinology and Metabolism Research Center (EMRC), Vali‐Asr Hospital, Imam Khomeini Hospital Tehran Iran; ^2^ School of Pharmacy and Pharmaceutical Sciences, Islamic Azad University of Tehran Medical Sciences Tehran Iran; ^3^ Faculty of Pharmacy, Ayatollah Amoli Branch, Islamic Azad University Amol Iran; ^4^ School of Medicine, Shahid Beheshti University of Medical Sciences Tehran Iran; ^5^ Universal Scientific Education and Research Network (USERN), Tehran Iran; ^6^ School of Medicine, Tehran University of Medical Sciences Tehran Iran; ^7^ Marie‐Josée and Henry R. Kravis Center for Cardiovascular Health at Mount Sinai Fuster Heart Hospital New York New York USA

**Keywords:** insulin resistance, lipid accumulation product, meta‐analysis, metabolic syndrome, polycystic ovary disease

## Abstract

**Background:**

Lipid accumulation product (LAP) index is a measure of lipid toxicity based on triglyceride and waist circumference. The aim of the current study is to explore the relationship between LAP index and metabolic syndrome (MetS) among women with polycystic ovary syndrome (PCOS) in a systematic review and meta‐analysis.

**Methods:**

Web of Science, Embase and PubMed online databases were systematically searched for studies investigating the relationship between LAP index and MetS in PCOS. Ten observational studies, including 12 populations with 2957 individuals, were identified for analysis. Mean difference and bivariate diagnostic test accuracy (DTA) meta‐analyses were performed.

**Results:**

A significant LAP index difference of 49.17 was found in women with PCOS with and without MetS (95% CI [40.57, 57.77]). By DTA meta‐analysis, the pooled sensitivity and specificity of LAP index for MetS detection were 87% (*I* ^2^ 78%, 95% CI [80%, 92%]) and 88% (*I* ^2^ 78%, 95% CI [83%, 92%]), respectively, with area under the curve of 0.94 (95% CI [0.91, 0.96]).

**Conclusion:**

The LAP index is an affordable, specific and sensitive marker for MetS and may be considered a pragmatic tool for MetS detection among patients with PCOS, particularly those with an insulin resistance endotype.

## Graphical abstract

Diagnostic performance of LAP index for detecting MetS in women with PCOS. AUC, area under the curve; LAP, lipid accumulation product; MD, mean difference; MetS, metabolic syndrome; PCOS, polycystic ovary syndrome.

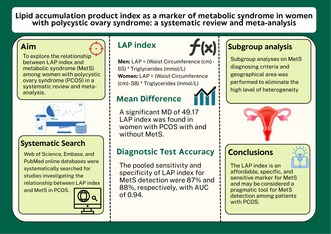

**Abbreviations**
AUC: area under the curve
BMI: body mass index
BP: blood pressure
DTA: diagnostic test accuracy
FBG: fasting blood glucose
HDL‐C: high‐density lipoprotein‐cholesterol
IDF: International Diabetes Federation
IQRs: interquartile ranges
IR: insulin resistance
JIS: joint interim statement
LAP: lipid accumulation product
MASLD: metabolic dysfunction‐associated steatotic liver disease
MD: mean difference
MetS: metabolic syndrome
NCEP‐ATP III: National Cholesterol Education Program‐Adult Treatment Panel III
PCOM: polycystic ovary morphology
PCOS: polycystic ovary syndrome
PRISMA: Preferred Reporting Items for Systematic Reviews and Meta‐analysis
QUADAS‐2: quality assessment of diagnostic accuracy studies 2
SD: standard deviation
sROC: summary receiver operating characteristic
T2D: type 2 diabetes
TG: triglycerides
WC: waist circumference

## 1 Background

Metabolic syndrome (MetS) is a medical condition defined by a cluster of cardiovascular risk factors with abnormal adiposity and insulin resistance (IR) as central drivers. Various diagnostic criteria are defined to detect MetS. Nevertheless, a MetS diagnosis generally requires the presence of three conditions out of the following five: elevated blood pressure (BP), fasting blood glucose (FBG), serum triglycerides (TG) level, reduced high‐density lipoprotein cholesterol (HDL‐C), and central obesity [1, 2]. Obesity occurs with excess adiposity defined by a body mass index (BMI) > 30 kg/m^2^ (> 5 kg/m^2^ in Asians) and leads to insulin resistance, hyperglycemia, and cardiovascular disease, imposing a significant healthcare burden globally [3]. Various lifestyle interventions targeting healthy dietary patterns, physical activity, sleep hygiene, among others, lead to a substantial reduction in the risk of MetS development, but implementation tactics have been challenging, especially in low‐to‐middle income and other vulnerable populations [4, 5, 6, 7]. Based on the DECODE study, which includes data from nine European countries, the age‐standardised prevalence of MetS was 15.7% and 14.2% in men and women, respectively [8]. In the United States, the prevalence of MetS is considerable—35% in the general population and nearly 50% in those aged 65 years and older [9]. MetS increases the incidence of type 2 diabetes (T2D) and cardiovascular events by two‐ and fivefold, respectively [10]. Furthermore, MetS may lead to other chronic diseases such as cancer, neurodegenerative diseases, reproductive disorders, metabolic dysfunction‐associated steatotic liver disease (MASLD), dyslipidemia and atherosclerosis [11].

Polycystic ovary syndrome (PCOS) is one of the most common endocrine disorders, affecting nearly 20% of women of reproductive age, according to the 2003 Rotterdam consensus [12, 13]. Although the exact cause of PCOS remains unclear, hormonal irregularity, inflammation, IR and hyperandrogenism play substantial roles in causing abnormal folliculogenesis and increasing risks for endometrial cancer and T2D [14]. On presentation, phenotypic signs of PCOS include ovulatory dysfunction, ovaries with polycystic morphology and hyperandrogenism. One mechanistic endotype of PCOS includes metabolic aspects such as abnormal adiposity (including obesity), hyperinsulinemia and IR [15]. Notably, IR occurs in 50%–90% of women with PCOS and is associated with MetS [16]. In fact, women with PCOS have a higher prevalence of MetS compared to age‐matched women without underlying PCOS [17, 18].

Excess fat can be stored as visceral fat or ectopic fat, which are significant contributors to metabolic and cardiovascular disturbances [19]. Visceral fat is accumulated within the abdominal cavity and is associated with IR, T2D and CVD, while ectopic fat is stored in abnormal locations such as the heart, liver, pancreas, kidney and muscles, which can lead to systemic inflammation, IR, organ dysfunction and cardiometabolic‐based chronic disease [20, 21, 22]. The lipid accumulation product (LAP) index is an adiposity marker with greater predictive value than BMI and is calculated by just using serum TG levels and waist circumference (WC) with the following formula [23].

$$\begin{array}{c} \mathrm{LAP}\;\text{index}:\;\text{Women}:\left({\mathrm{WC}}^{\mathrm{cm}}–58\right)\times {\mathrm{TG}}^{\text{mmol}/L},\\ \mathrm{men}:\left({\mathrm{WC}}^{\mathrm{cm}}–65\right)\times {\mathrm{TG}}^{\text{mmol}/L}. \end{array}$$

This marker can be used to detect MASLD, MetS and T2D [24, 25, 26]. Individuals diagnosed with both MetS and PCOS have greater IR and hyperandrogenism, with the potential of progressing to more severe conditions [18, 27], highlighting the importance of detecting MetS in women with PCOS. Although the LAP index is considered as a reliable index for detecting MetS in the general population [28], there is no consensus regarding its effectiveness in detecting MetS in women with PCOS. Accordingly, the aim of this study is to evaluate the LAP index as a marker for detecting MetS among women with PCOS.

## 2 Methods

This systematic review and meta‐analysis is conducted based on the Preferred Reporting Items for Systematic Reviews and Meta‐analysis (PRISMA) statement 2020 [29]. The article's protocol was registered in the International Prospective Register of Systematic Reviews (PROSPERO): CRD42024566516.

### 2.1 Study Selection

#### 2.1.1 Eligibility Criteria

Only observational studies involving human subjects were included based on the following PICO criteria:

- Population (P): Adult women (≥ 18 years of age) diagnosed with both PCOS and MetS.
- Intervention (I): Assessment using the LAP index.
- Comparison (C): Adult women (≥ 18 years) with PCOS but without a diagnosis of MetS.
- Outcome (O): The comparison of mean LAP between patients with and without MetS, and the diagnostic accuracy of the LAP index for identifying MetS among women with PCOS.

This systematic review includes original, observational, and peer‐reviewed studies. Studies reporting the means and standard deviations (SDs) of LAP index in patients with and without MetS, and/or sensitivity and specificity of LAP index for detecting MetS among women with PCOS were eligible.

#### 2.1.2 Exclusion Criteria

Papers with inadequate information such as sample size for participants with and without MetS, or those diagnosed with PCOS, were not eligible for this study. Additionally, preprint papers, review articles, editorial letters, reports, randomised control trials, dissertations, and book chapters were not included due to concerns regarding lack of peer review and quality. Studies performed on paediatrics and adolescents (< 18 years old) were also excluded.

### 2.2 Search Strategy

Web of Science, Embase and PubMed databases were searched systematically until November 2024. Search strings were related to the LAP index, MetS and PCOS (Supporting Information). Duplicate results were removed using Zotero software. According to the criteria mentioned above, two independent authors (A.M. and D.A.) screened the title/abstract, and then for the remaining studies, reviewed the full texts. Regarding conflict, the reviewers discussed reaching an agreement. If unable, the third reviewer resolved disagreements (S.A.S.).

This systematic search was performed without any language restrictions.

### 2.3 Data Extraction

Two independent collaborators extracted data (S.A.A. and A.N.) from included studies. If they could not reach agreement regarding an item, the third reviewer resolved the disagreement (S.A.S.).

The data extracted from articles were the first author's name, publication year, design of the study, location of the study, important inclusion and exclusion criteria, total case number, total control number, mean and SD of LAP index for case and control groups, and sensitivity/specificity, area under the curve (AUC), and cut‐off values if reported. Additionally, for performing meta‐analysis, the LAP index values reported in mg/dL were converted to mmol/L.

### 2.4 Quality Assessment

The quality of the included studies was evaluated using the quality assessment of diagnostic accuracy studies 2 (QUADAS‐2) tool. This tool assesses studies across four different domains: patient selection, index test, reference standard, and flow/timing. Each domain consists of three signalling questions, scored as yes/no/unclear, indicating low risk of bias, high risk of bias, or inability to assess bias due to insufficient information, respectively. Additionally, QUADAS‐2 addresses applicability concerns through three different domains: patient selection, index test, and reference standard, with each domain also receiving low/high/unclear scores based on studies' information. Any disagreements were resolved through discussions with other reviewers. The graphical display template from the University of Bristol was used to visually represent the QUADAS‐2 assessment [30].

### 2.5 Publication Bias

Funnel plot and Egger's test with a significance level of *p* < 0.05 were used to assess publication bias.

### 2.6 Statistical Analysis

Meta‐analyses were conducted using STATA version 14 with the “Metan” and “Midas” commands. The random‐effects model was employed. For studies reporting means and SDs of the LAP index, a mean difference (MD) meta‐analysis was performed. In addition, an influence test was performed to evaluate the impact of each study on the overall results of the MD meta‐analysis. For studies reporting interquartile ranges (IQRs) and medians, SD was calculated using the following formula calculated by Wan et al. [31]:

$$\mathrm{SD}=\left(q3-q1\right)/1.35$$

In addition, for performing the diagnostic test accuracy (DTA) meta‐analysis, studies with sensitivity and specificity values for detection of MetS with LAP index were included. Bivariate meta‐analysis was conducted to calculate pooled sensitivity and specificity; this model is applied when different cut‐offs are reported in studies. Additionally, the accuracy of LAP index for detection of MetS was investigated by drawing the sROC and calculating the pooled AUC. The heterogeneity of the meta‐analysis was evaluated with the Cochrane *Q* test and *F* test. Significant heterogeneity of data was defined as *I* ^2^ > 50% or a significant Cochrane‐*Q* test (*p* < 0.10). Subgroup analyses were conducted to explore potential confounding factors including MetS diagnosing criteria and geographical area.

## 3 Results

### 3.1 Study Characteristics

**FIGURE 1:**
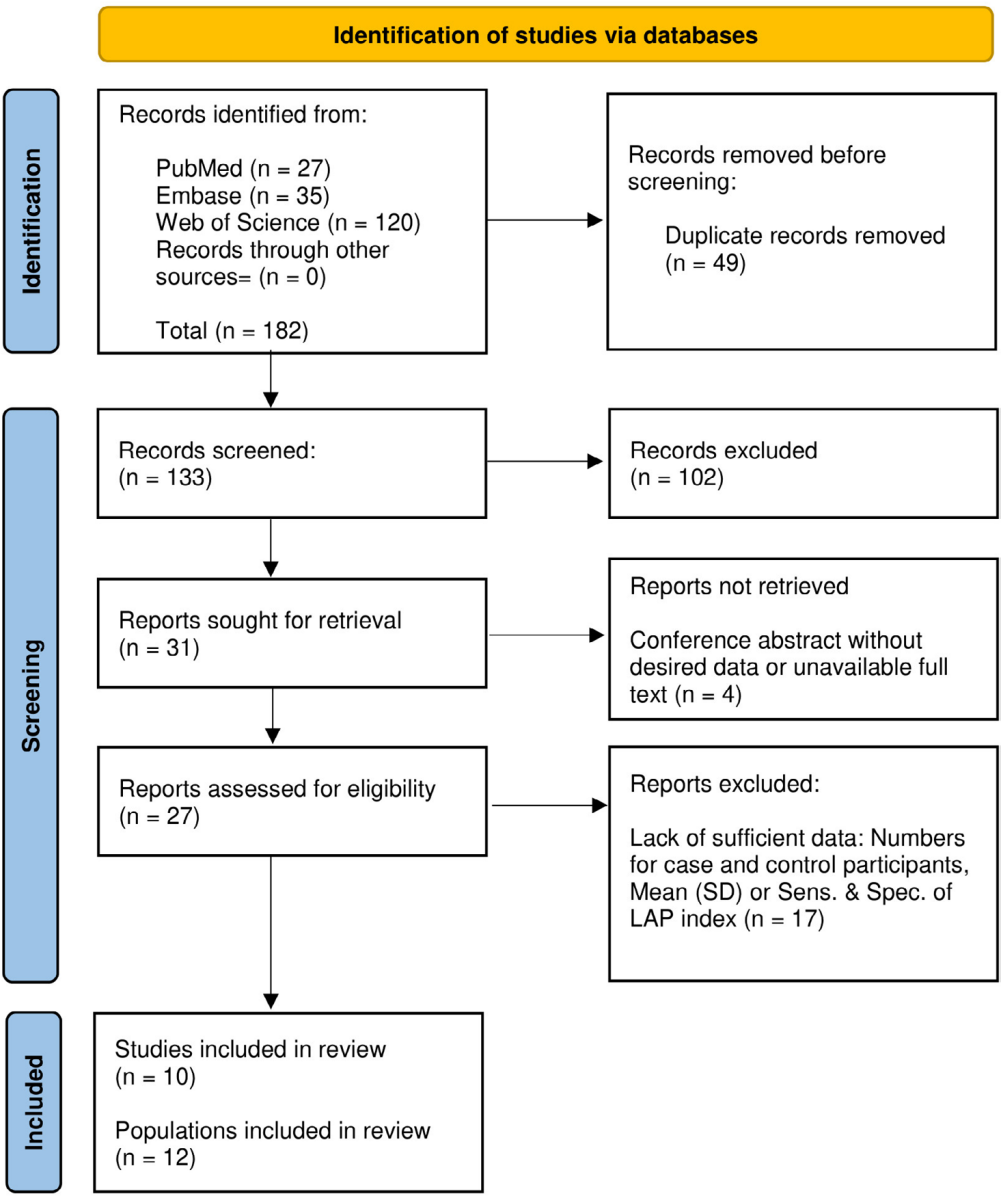
PRISMA flow diagram of included studies. Abbreviations: LAP – lipid accumulation product; PRISMA – preferred reporting items for systematic reviews and meta‐analyses; Sens – sensitivity; SD – standard deviation; Spec – specificity.

The systematic search result of the study is shown in Figure 1 in a stepwise pattern. Initially, three major databases including Embase, Web of Science and Pubmed were searched with the related search string. After removing the duplicated results, 133 unique records remained. Moreover, 102 articles were excluded by title/abstracts as they were not related to the intention of this systematic review. In the following step, the remaining articles were evaluated based on their full text. Based on the eligibility criteria, studies performed on children and adolescents were excluded. Finally, 10 eligible cross‐sectional studies providing information on 12 distinct populations, reporting either means of LAP index in patients with MetS and without MetS, or sensitivity and specificity of LAP index for detection of MetS, were determined and included in the study.

Ten studies with 2957 participants with PCOS were included in this meta‐analysis. Of these, 671 had MetS and 2286 did not have MetS. The effect size in included articles varied from 62 to 1083, and the participant age ranged from 18 to 40 years. National Cholesterol Education Program‐Adult Treatment Panel III (NCEP‐ATP III) (*N* = 6), International Diabetes Federation (IDF) (*N* = 5), and Joint Interim Statement (JIS) (*N* = 1) were the criteria for diagnosis of MetS used by included populations (Table 1). All of them, except for three populations included in the study by W. Han et al. [36], showed significantly higher mean BMI in the case group (*p* < 0.001).

**TABLE 1:** Characteristics of the included articles.

Author, year	Country/continent	Study design	Age mean/age range	Total population (*N*)	MetS participants	MetS criteria	Mean case BMI (SD)	Mean control BMI (SD)	BMI *p*	LAP cut‐off	Mean LAP in case (SD)	Mean LAP in control (SD)	LAP OR (95% CI)	Sensitivity	Specificity
H. Banu [32]	Bangladesh	CS	18.0–24.0	62	52	NCEP‐ATP III	< 23 kg/m^2^	< 23 kg/m^2^	NR	23.24	NR	NR	1.124 (1.050–1.202)	0.900	0.731
S. Xiang [33]	China	CS	18–34	105	45	IDF	27.2 (2.2)	22.7 (1.7)	< 0.001	54.2	95.2 (28.9)	26.8 (12.8)	NR	0.933	0.967
Q. Yin [34]	China	CS	16–41	1083	124	IDF	NR	NR	NR	45.13	NR	NR	NR	0.880	0.884
D. Macut [35]	Europe	CS	25.01 (4.89)	222	36	NCEP‐ATP III	30.3 (3.8)	21.5 (3.1)	< 0.001	25.94	91.8 (16.7)	16.7 (15.4)	NR	0.810	0.910
W. Han [36]	China	CS	20–40	77	12	NCEP‐ATP III (normal weight)	22.59 (1.12)	22.10 (1.21)	0.190	42.5	54.74 (23.22)	19.8 (19.8)	1.21 (1.06–1.37)	0.917	0.969
W. Han [36]	China	CS	20–40	70	29	NCEP‐ATP III (overweight)	25.93 (1.05)	25.80 (1.14)	0.648	47.95	71.74 (54.64)	29.7 (28.89)	1.10 (1.037–1.162)	0.931	0.854
W. Han [36]	China	CS	20–40	95	68	NCEP‐ATP III (obese)	30.99 (2.28)	30.54 (1.98)	0.367	NR	80.55 (51.73)	53 (66.36)	1.02 (0.996–1.050)	NR	NR
Ma. G. Kaluzna [37]	Poland	CS	18–40	404	64	IDF	35.9 (8.1)	23.2 (7.1)	< 0.001	30.75	72.8 (13.5)	13.5 (19.1)	NR	0.968	0.817
Z. Naghshband [38]	India	CS	25.78 (5.00)	150	89	IDF	28.05 (5.22)	24.81 (4.80)	< 0.001	53	81.51 (37.2)	37.2 (17.65)	NR	0.680	0.800
S. A. Polyzos [39]	Greece	CS	26.1 (0.4)	314	77	IDF	36.8 (0.8)	30.1 (0.4)	< 0.001	NR	76.8 (4.9)	33.4 (1.4)	NR	NR	NR
F. R. Tehrani [40]	Iran	CS	18–45	175	31	JIS	NR	NR	NR	38	NR	NR	NR	0.74	0.91
R. A. Shreenidhi [41]	India	CS	28.11 (3.91)	200	44	NCEP‐ATP III	NR	NR	NR	48.06	NR	NR	NR	0.79	0.79

Abbreviations: BMI, body mass index; CI, confidence interval; CS, cross‐sectional; IDF, International Diabetes Federation; JIS, joint interim statement; LAP, lipid accumulation product; MetS, metabolic syndrome; NCEP‐ATP III, National Cholesterol Education Programme‐Adult Treatment Panel III; NR, not reported; SD, standard deviation.

### 3.2 Mean Difference

**FIGURE 2:**
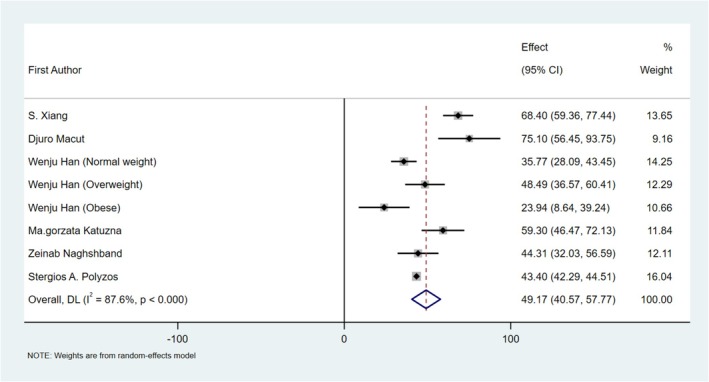
Forest plot showing the pooled mean difference in the LAP index between participants with and without MetS among participants with PCOS. Abbreviations: CI, confidence interval; LAP, lipid accumulation product; MetS, metabolic syndrome; PCOS, polycystic ovary syndrome.

All included studies in the MD meta‐analysis [33, 35, 36, 37, 38, 39] reported a significant association between MetS and LAP index in participants with PCOS. The random effect meta‐analysis demonstrated a significantly higher pooled mean of LAP index in those with MetS by 49.17 units (*I* ^2^ 87.6%; 95% CI [40.57, 57.77]; *p* < 0.001) (Figure 2).

The pooled effects remained stable after the influence test was performed, illustrating that no specific study significantly impacted the overall results of the MD meta‐analysis. Subgroup meta‐analysis based on geographical area and MetS diagnostic criteria was performed; however, it did not completely eliminate the source of heterogeneity (Table 2).

**TABLE 2:** Subgroup meta‐analysis by MetS diagnosing criteria and geographical area.

Subgroup analysis	Number of populations	Pooled mean difference	*I* ^2^ (*p*)	Cochrane's Q *p*
MetS diagnosing criteria				
IDF	4	53.59 (40.00–67.18)	91.3%	(< 0.001)
NCEP‐ATP III	4	44.82 (28.20–61.44)	85.6%	(< 0.001)
Geographical area				
China	4	44.59 (25.94–63.25)	92.2%	(< 0.001)

Abbreviations: CI, confidence interval; IDF, International Diabetes Federation; MetS, metabolic syndrome; NCEP‐ATP III, National Cholesterol Education programme‐Adult Treatment Panel III.

### 3.3 DTA of LAP Index

Nine studies were included in the total DTA meta‐analysis [32, 33, 34, 35, 36, 37, 38, 40, 41]. Distinct cut‐off values for the LAP index were reported in included studies. The correlation between the specificity and sensitivity was evaluated, which was not significant, demonstrating that different cut‐offs had no significant impact on the DTA meta‐analysis.

**FIGURE 3:**
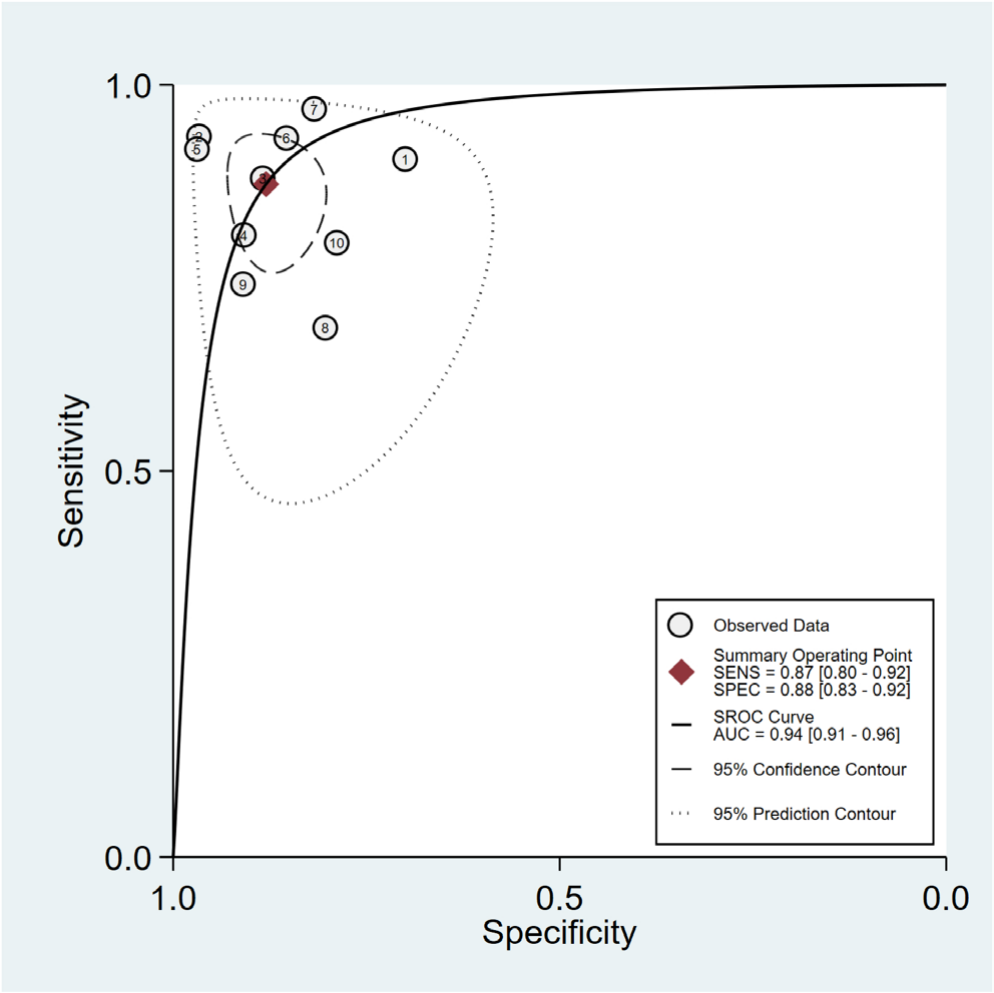
sROC curve for the accuracy of the LAP index for detection of MetS in patients with PCOS. Numbers are included studies with reported sensitivity and specificity; 95% confidence contour and prediction contour are depicted with dashed and dotted lines, respectively. Abbreviations: AUC, area under the curve; LAP, lipid accumulation product; MetS, metabolic syndrome; PCOS, polycystic ovary syndrome; SENS, sensitivity; SPEC, specificity; sROC, summary receiver operating characteristic.

A bivariate DTA meta‐analysis was performed, and joint sensitivity and specificity were 87% (*I* ^2^ = 78%; 95% CI [80%, 92%]) and 88% (*I* ^2^ = 78%; 95% CI [83%, 92%]), respectively. Moreover, the summary receiver operating characteristic (sROC) area under the curve (AUC) of 0.94 (95% CI [0.91, 0.96]) indicates that the LAP index has high accuracy for MetS detection in populations with PCOS (Figure 3).

The pooled diagnostic odds ratio was 50 (*I* ^2^ = 78%; 95% CI [25–98]), indicating that the probability of having MetS in participants with high LAP index is 50‐fold higher than in participants with low LAP index in PCOS.

### 3.4 Methodological Quality

The studies' risk of bias evaluation based on the QUADAS‐2 tool is depicted in Supporting Information. Two studies were classified at high risk of bias, while the other eight studies were classified at low risk of bias, despite some shortcomings in the patient selection criteria. The origin of the risk of bias in the included studies was mainly from patient selection criteria and some unclarity in the index test, as well as reference standard criteria.

### 3.5 Publication Bias

**FIGURE 4:**
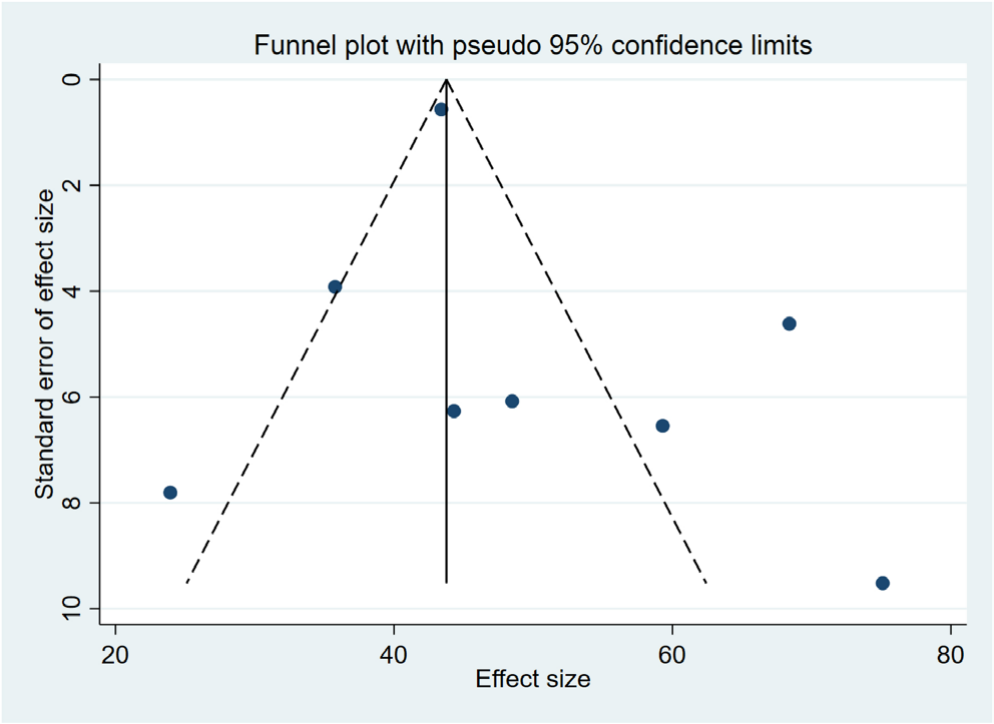
Funnel plot assessing publication bias among the included studies. Dots represent studies; dashed lines represent pseudo 95% confidence limits.

After inspection of funnel plots and Egger's test, no significant publication bias was present (*p* = 0.258) (Figure 4).

## 4 Discussion

To the best of our knowledge, this meta‐analysis is the first to demonstrate the association between the LAP index and MetS in patients with PCOS. The current study, encompassing 10 studies and 12 distinct populations, revealed that the LAP index serves as a highly effective marker for the detection of MetS in women with PCOS. As demonstrated by MD meta‐analysis, all included studies exhibited higher mean LAP values in individuals with MetS compared to controls, emphasising the potential clinical relevance. The DTA meta‐analysis resulted in high aggregated sensitivity and specificity of the LAP index for the diagnosis of MetS with a high AUC. These results highlight the efficacy of LAP as a reliable and precise marker for detecting MetS in patients with PCOS, supporting application in clinical settings for early diagnosis and management.

Metabolic abnormalities associated with PCOS can be exacerbated by obesity, but they can also be apparent in the absence of obesity, leading to the development of MetS [42, 43, 44, 45]. Dyslipidemia plays a substantial role in MetS pathophysiology and increases the risk of CVD independent of an increased BMI [46]. Although the results of this study indicate that populations diagnosed with MetS and PCOS had significantly higher BMI compared to their respective controls, non‐obese individuals with PCOS still have a risk of developing MetS, as demonstrated by W. Han et al. and Banu et al. [32, 36]. Additionally, W. Han et al. indicated that the LAP index has greater diagnostic value in normal‐weight populations compared to overweight and obese populations, as evidenced by significantly higher OR values [36].

Dyslipidemia is also associated with PCOS, with reports indicating a prevalence of 70% among women with PCOS in the United States; while in the Chinese PCOS population, the prevalence of dyslipidemia ranges from 41.3% to 53.1% [47]. MetS is a common finding in the general population, with prevalence rates ranging from 8.2% to 47.3% in various countries, depending on the diagnostic criteria [37, 48, 49, 50]. In contrast to the NCEP‐ATP III criteria, the IDF criteria require central obesity [51, 52]. A subgroup meta‐analysis is performed in the current study to evaluate studies with the same MetS diagnostic criteria together.

PCOS is characterised by a variety of phenotypic features such as oligo‐anovulation, hyperandrogenism and characteristic polycystic ovary morphology (PCOM). These and other features are clustered into four distinct groups by the Rotterdam criteria: phenotype A (hyperandrogenism, oligo‐anovulation and PCOM), phenotype B (hyperandrogenism and oligo‐anovulation), phenotype C (hyperandrogenism and PCOM) and phenotype D (oligo‐anovulation and PCOM) [12, 53]. Recent studies have emphasised the clinical importance of PCOS phenotypes and their mechanistic endotypes, especially those with abnormal metabolic features. For instance, a higher risk of IR and MetS development is associated with phenotypes A and B, while phenotype D without hyperandrogenism does not require the same attention regarding metabolic disturbances [54, 55]. However, included studies did not take these phenotypes into account in their analyses.

Recent investigations highlighted the substantial association between MetS traits and IR endotypes of PCOS. Women with these IR endotypes are at increased risk of developing MetS [56, 57]. Lifestyle modifications with the aim of improving IR in these individuals have been shown to be a beneficial course of management. In addition, pharmacotherapy including metformin has been demonstrated to reduce the metabolic risks associated with these phenotypes [58, 59]. Accordingly, initial interventions addressing IR can lead to enhanced reproductive health and decreased cardiometabolic risk; therefore, routine evaluation of metabolic disturbances in women with PCOS is recommended [53, 57, 60].

The LAP index was initially introduced as a more effective tool than BMI for detecting adult cardiovascular risks. In a study by Vieira et al. [61], a statistically significant difference was found in anthropometric and biochemical markers for CVD risk among individuals with higher LAP values compared to those with low LAP values. This index combines two specific measurements, WC and TG, to take into account anatomical and physiological changes associated with visceral fat deposition [62]. An increase in LAP may indicate excessive lipid accumulation in ectopic tissues, including the liver, skeletal muscles, heart, blood vessels, kidneys, and pancreas, as well as in visceral fat [63, 64]. In addition, LAP is associated with a variety of medical conditions. Lin et al. [65] demonstrated that LAP could be used as a predictor of early‐onset T2D. In a meta‐analysis by Witarto et al. [28], the LAP index was found to be a cost‐effective and accurate tool, among other adiposity indices, for the diagnosis of MetS in the general population. Moreover, Banu et al. [32] demonstrated that LAP exhibited predictive associations with blood pressure, age and low‐density lipoprotein cholesterol levels and served as a moderately effective indicator of IR in lean patients with PCOS.

Studies demonstrated an 11‐fold increased risk of MetS development in women with PCOS compared to a healthy control group, which highlights the importance of MetS screening and diagnosis in PCOS populations [48, 66]. Hyperlipidaemia and IR are the underlying abnormalities responsible for this increased risk, which ultimately could lead to CVD if not adequately addressed [13, 27]. Accordingly, early diagnosis of MetS, with pragmatic markers including LAP, is crucial in PCOS management not only to reduce cardiovascular comorbidities but also to prevent associated health burdens. Increased LAP index is actionable with intensive lifestyle change or cardiometabolic pharmacological interventions.

## 5 Strength and Limitations

The strengths of this study include comprehensiveness and the use of replicable methods for searching the literature. In addition, ours is the first systematic review and meta‐analysis on the LAP index and MetS in PCOS. Also, this study proposes the usefulness of the LAP index as a low‐cost and pragmatic method for detecting MetS in women with PCOS.

There are limitations in this study. Laboratory measures were determined in different facilities with distinct equipment, which could cause inconsistencies. Additionally, included studies did not investigate the MetS and PCOS association based on PCOS phenotypes; thus, this study could not evaluate this association to a PCOS‐phenotype level. Moreover, the differences in diagnostic criteria for MetS and PCOS in different studies could lead to some discrepancies in this study's final result, which may be the source of increased heterogeneity.

Although subgroup analysis on MetS diagnostic criteria and geographical area was performed, this increased heterogeneity was not fully eliminated. Some other sources of heterogeneity should be explained, which could not be evaluated by subgroup analysis, including the presence of underlying disease, duration of MetS and lifestyle factors including alcohol intake, physical activity and smoking. Finally, different cut‐offs presented in each study could lead to some discrepancies in the final results.

## 6 Conclusion

This study supports the use of the LAP index as a pragmatic marker of MetS in PCOS. Increased detection of MetS in the PCOS population can potentially identify patients at higher risk for cardiovascular disease, prompting early preventive care that can lead to decreased cardiometabolic complications and clinical burdens. Finally, further studies evaluating the association of MetS and PCOS based on PCOS phenotypes are recommended.

## Author Contributions

S.A.S.: conceptualisation, data curation, investigation, methodology, project administration, writing – original draft, and writing – review and editing. S.A.N.: conceptualisation, formal analysis, and visualisation. S.A.A.: data curation and investigation. A.M.: data curation and investigation. D.A.: Data curation and investigation. S.H.: investigation and methodology. F.E.A.: writing – original draft. J.I.M.: supervision and writing – review and editing. A.E.: supervision, validation, writing – review and editing.

## Ethics Statement

The authors have nothing to report.

## Consent

The authors have nothing to report.

## Conflicts of Interest

Dr. Jefferey I. Mechanick received honoraria from Abbott Nutrition and serves on the Advisory Boards of Abbott Nutrition, Aveta Life and Twin Health. All other authors declare no conflicts of interest.

## Supporting information


Data S1.

## Data Availability

The datasets used and/or analysed during the current study are available from the corresponding author on reasonable request.
