# Supplementary material for: Lipid Accumulation Product Index as a Marker of Metabolic Syndrome in Women With Polycystic Ovary Syndrome: A Systematic Review and Meta‐Analysis
Source: Endocrinol Diabetes Metab. 2025 Sep 4;8(5):e70078. doi: 10.1002/edm2.70078 (PMC12411561; doi:10.1002/edm2.70078)
Supplement: Supplementary file 1 — Data S1. [file EDM2-8-e70078-s001.docx]

**Supplementary Materials**

**Search Strings:**

Pubmed:

((("metabolic syndrome"[MeSH Terms]) OR ("metabolic syndrome"[All Fields] OR "syndrome X"[All Fields] OR "X syndrome"[All Fields] OR "insulin resistance syndrome"[All Fields])) AND ((lipid accumulation product) OR (LAP index))) AND (((Polycystic Ovary Syndrome) OR (PCOS)) OR "Polycystic Ovary Syndrome"[MeSH Terms])

Embase:

('metabolic syndrome' OR 'syndrome x' OR 'insulin resistance syndrome') AND ('lipid accumulation product index' OR 'lap index') AND ('ovary plycystic disease' OR 'pcos' OR 'polycystic ovary syndrome')

Web of Science:

(Polycystic ovary syndrome(All Fields) or PCOS(All Fields)) AND (Lipid accumulation product(All Fields) or LAP index(All Fields)) AND (metabolic syndrome(All Fields) or syndrome x(All Fields) or Insulin resistance syndrome(All Fields) or X syndrome(All Fields))

**QUADAS-2**

Risk of Bias

|  | **PATIENT SELECTION** | **INDEX TEST** | **REFERENCE STANDARD** | **FLOW AND TIMING** |
| --- | --- | --- | --- | --- |
| H. Banu 2022 | High | Low | Low | Low |
| S. Xiang 2012 | Low | Low | Low | Low |
| Q. Yin 2021 | Low | Low | Unclear | Low |
| D. Macut 2016 | High | Low | Low | Low |
| W. Han 2024 | Low | Low | Low | Low |
| Ma. g. Katuzna 2022 | Low | Low | Low | Low |
| Z. Naghshband 2021 | High | Unclear | Low | Low |
| S. A. Polyzos 2014 | High | Low | Low | Low |
| F. R. Tehrani 2014 | High | Low | Low | Low |
| R. A. Shreenidhi 2024 | Low | Unclear | Low | Low |

Applicability Concerns

|  | **PATIENT SELECTION** | **INDEX TEST** | **REFERENCE STANDARD** |
| --- | --- | --- | --- |
| H. Banu 2022 | Low | Low | Low |
| S. Xiang 2012 | Low | Low | Low |
| Q. Yin 2021 | Low | Low | Unclear |
| D. Macut 2016 | Low | Low | Low |
| W. Han 2024 | Low | Low | Low |
| Ma. g. Katuzna 2022 | Low | Low | Low |
| Z. Naghshband 2021 | Low | Low | Low |
| S. A. Polyzos 2014 | Low | Low | Low |
| F. R. Tehrani 2014 | High | Low | Low |
| R. A. Shreenidhi 2024 | Low | Low | Low |
